# Level of eHealth Literacy and Its Associations With Health Behaviors and Outcomes in Chinese Older Adults: Cross-Sectional Analysis of Baseline Data From a Large-Scale Community Project

**DOI:** 10.2196/74110

**Published:** 2026-01-30

**Authors:** Siu Long Chau, Wanjia He, Tzu Tsun Luk, Sophia Siu Chee Chan

**Affiliations:** 1 School of Public Health, Li Ka Shing Faculty of Medicine, University of Hong Kong, Metro South Tower 1, 39 Wong Chuk Hang Road, Wong Chuk Hang, Hong Kong, China (Hong Kong), 852 39176610; 2 Alice Lee Centre for Nursing Studies, Yong Loo Lin School of Medicine, National University of Singapore, Singapore

**Keywords:** eHealth literacy, older adults, health behaviors, health-related outcomes, healthy aging

## Abstract

**Background:**

eHealth literacy is important for older adults to be able to seek and evaluate online health information. However, there is a scarcity of large-scale data on their eHealth literacy levels, particularly among the oldest older individuals (aged >75 years) in unique, high-income Asian regions such as Hong Kong. A comprehensive understanding of how eHealth literacy is associated with specific health behaviors, mental well-being, and physical health outcomes in this population is lacking.

**Objective:**

This study aims to assess the level of eHealth literacy and its associations with health behaviors and health-related outcomes among older adults in Hong Kong.

**Methods:**

We conducted a cross-sectional analysis of baseline data from the Generations Connect Project. This is an ongoing large-scale community-based project, where we trained university students to conduct home visits and assess the health status of older adults (N=6704) in Hong Kong. eHealth literacy was measured using the eHealth Literacy Scale (eHEALS; score: 8‐40). Health behavior measurements included physical activity levels (metabolic equivalent of task minutes per week) and smoking, drinking, and eating habits. Mental well-being was measured using the World Health Organization-Five Well-Being Index (percentage score: 0‐100) and UCLA 3-item Loneliness Scale (score: 3‐9). Physical health was assessed on the basis of self-reported medical diagnosis of noncommunicable diseases (yes/no), including hypertension, diabetes, cardiovascular disease, and stroke. Adjusted unstandardized coefficients (b) and odds ratios (ORs) were calculated to determine the associations between variables.

**Results:**

Among the 6704 participants (mean age 77.8, SD 7.0 years), the mean eHEALS score was 18.2 (SD 10.2), and 44.1% (2897/6566) of the participants had inadequate eHealth literacy (score: 8‐15.99). Increasing age (adjusted b −0.32, 95% CI −0.35 to −0.28; *P*<.001), support from the Comprehensive Social Security Assistance Scheme (adjusted b −1.49, 95% CI −2.04 to −0.95; *P*<.001), and living in public housing (adjusted b −1.60, 95% CI −2.69 to −0.50; *P*=.004) were associated with a lower eHEALS score. Participants with moderate eHealth literacy (score: 24‐31.99) were less likely to be current smokers (adjusted OR 0.60, 95% CI 0.38-0.95; *P*=.04), more physically active (adjusted b 39.83, 95% CI 2.04-77.62; *P*=.04), more likely to be community health center members (adjusted OR 1.52, 95% CI 1.30-1.77; *P*<.001) and to have healthy diets (adjusted b 0.034, 95% CI 0.006-0.063; *P*=.04), and less likely to have a medical diagnosis of diabetes (adjusted OR 0.73, 95% CI 0.62-0.85; *P*<.001). Moreover, they had a higher score on the World Health Organization-Five Well-being Index (adjusted b 2.89, 95% CI 1.42-4.36; *P*<.001) and a lower score on the UCLA 3-item Loneliness Scale (adjusted b −0.26, 95% CI −0.37 to −0.15; *P*<.001).

**Conclusions:**

The level of eHealth literacy was low among older adults in Hong Kong. eHealth literacy was associated with positive health behaviors and health-related outcomes. Interventions are warranted to boost their eHealth literacy in the future.

## Introduction

Older adults (aged >60 years) accounted for 5% of the global population in the last few decades, and this estimate is projected to rise to 16% by 2050 [1]. In China, the population aged ≥80 years reached 36 million in 2020 and will increase to 115 million in 2050 [2]. This global demographic shift toward an older population is accompanied by an increased burden of noncommunicable diseases (NCDs) [3] and mental illnesses [4], which places immense strain on public health care systems. More than 20% of older adults in the world have 1 or more mental illnesses [4], and the prevalence increases with population size. In 2017, 8.6% of older adults in Hong Kong (N=4368) had depression and anxiety [5]. Mental illnesses among older people remained underrecognized and undertreated, which accounted for 10.6% of the total loss of disability-adjusted life years in older adults [6]. In response, and in line with the World Health Organization’s call for primary health care reform [7], there is an urgent need for innovative strategies to support healthy aging. The rapid development of digital technologies, accelerated by the COVID-19 pandemic, offers a key opportunity. Digital tools have the potential to support older adults’ intrinsic capacity, promote active and healthy aging, and alleviate the economic burden on health care sectors, making them an increasingly recognized priority in geriatric care [8].

The emergence of digital health offers a new avenue for improving individuals’ quality of life. Rapid advances in information and communications technology contribute to the increasing innovation and upgrading of health service modes [9]. Digital health enables low-cost, timely health care services for older adults and supports their physical and mental health by providing access to reliable health information, self-monitoring tools for chronic conditions, and social connection platforms [9]. Although accessing online medical services and information is convenient, the quality of online information varies [10]. Older adults need eHealth literacy to navigate and evaluate the accuracy of medical information, especially in the context of primary health care reform in Hong Kong, where the government is committed to enhancing digital primary health care services to shift the emphasis from tertiary to primary care.

eHealth literacy is defined as the ability of an individual to find and evaluate health information from online platforms and apply health-related knowledge to address health problems [11]. Although smartphone ownership has increased among older adults [12], they often possess lower technological skills than the younger generations and are consequently less likely to use these devices to search for health information [12]. eHealth literacy impacts how older adults search for health information online, which might influence health-related decisions [12]. With the rapid and continuous development of eHealth resources, it is crucial to assess older adults’ eHealth literacy and examine its association with health-related outcomes.

Previous studies suggested that higher eHealth literacy was associated with some healthy aging components (eg, less cognitive impairment and functional limitation) in older adults [13]. However, database searches in PubMed and CINAHL (using keywords such as “eHealth literacy,” “older adults,” “aging population,“ “health behavior,” “smoking,” and “physical activity”) suggest that studies on the association of eHealth literacy with specific health behaviors (eg, smoking, drinking, and physical exercise) and health status are scarce. Only 1 recent meta-analysis showed a positive association between eHealth literacy and general health-related behaviors (overall estimate of the correlation: 0.31, 95% CI: 0.25‐0.34), with a similar effect size observed in older adult populations [14]. Another systematic review showed that higher eHealth literacy was associated with better health knowledge and attitude in older adults, but the associations with physical and psychosocial outcomes were still inconsistent [15]. The relationships between eHealth literacy and specific health-related outcomes (eg, mental and physical well-being) need to be assessed to further explore their underlying mechanisms. In addition, studies on digital health often recruit participants from more accessible, community-dwelling populations [16], potentially underrepresenting the most vulnerable, such as homebound or socioeconomically disadvantaged older adults, who may face the greatest digital divide.

While existing research provides a foundational understanding of eHealth literacy, several critical gaps remain, limiting the development of effective, evidence-based interventions for older adults. First, eHealth literacy levels are known to vary significantly across different sociocultural and demographic contexts, with studies in the United States [16], South Korea [17], and Norway [18] reporting higher average scores than those reported in mainland China [19]. However, there is a scarcity of large-scale, representative data from Hong Kong, a unique high-income region characterized by extreme longevity, high digital penetration, and a government-led push for digital primary care reform. Second, much of the existing literature focuses on younger older populations (eg, those aged 60-65 years); however, the challenges and capabilities of the oldest older individuals (ie, those aged ≥75 years) may differ substantially. Third, while a study has confirmed a general positive association between eHealth literacy and composite health-related behaviors [14], there is a lack of research examining the associations between eHealth literacy and a comprehensive suite of specific, modifiable behaviors (eg, smoking, physical activity, and diet) and health service use. Finally, outcomes are often studied in silos. A holistic understanding requires a simultaneous investigation of the links between eHealth literacy and a broad spectrum of outcomes, including health behaviors, mental well-being, social connectedness, and physical health status.

To address these gaps, this study aimed to (1) assess the level of eHealth literacy in a large, community-based sample of underprivileged older adults in Hong Kong, with a particular focus on the oldest older population and (2) comprehensively examine the associations of eHealth literacy with specific health behaviors, mental health outcomes (well-being and loneliness), and physical health outcomes (prevalence of multiple NCDs).

## Methods

### Study Design

This study used a cross-sectional design, using baseline data from the Generations Connect Project. The parent project is an ongoing, large-scale, community-based, quasi-experimental pre-post study that trained over 1000 nursing students to provide home visits and perform a baseline health assessment and to deliver a health intervention (face-to-face and digital) to older adults in Hong Kong. The present study focused on the analysis of the baseline data collected before any intervention took place.

Older adults were recruited through collaborations with 20 local nongovernmental organizations covering 18 districts across Hong Kong. Between November 2022 and December 2024, we recruited participants aged ≥65 years who could read and communicate in Chinese and were cognitively and physically capable of understanding and answering survey questions. Older adults who were bedbound and had a history of mental illnesses were excluded. The exclusion of older adults with self-reported mental illness was to minimize potential confounding effects on the mental health outcomes measured in this study. In contrast, older adults with physical limitations (eg, visual or hearing impairments) were not excluded, as these conditions are prevalent in the target population, and their inclusion is crucial for the generalizability of our findings regarding the digital divide among the oldest older population. We recruited University of Hong Kong students to be student ambassadors through open recruitment via internal university emails and on-campus promotional materials (eg, posters and flyers) as well as selected curriculum (medical, nursing, dental, and common core courses) integration. Before making a home visit, all student ambassadors received a 3-hour training to ensure the intervention’s fidelity and data quality during the data collection process. Details of the training session and intervention are reported elsewhere [20].

### Ethical Considerations

Ethical approval was sought from the Institutional Review Board of the University of Hong Kong/Hospital Authority Hong Kong West Cluster (IRB/REC reference: UW 22‐693). The institutional review board also allowed secondary analysis without additional consent. The reporting of this study strictly followed the STROBE (Strengthening the Reporting of Observational Studies in Epidemiology) guidelines (Checklist 1). The funder of the study had no role in the study design, data collection, data analysis, data interpretation, or writing of the report.

Informed consent was obtained using comprehensive information sheets and verbal explanations, and signed consent forms were obtained from participants before any procedures began. The consent form included information about the study’s purpose and procedures and the participant’s right to withdraw at any time without penalty.

Participants enrolled in the study received gift bags worth HK $50 (US $6.44) as an incentive during the initial student visits.

All data collected during the study were deidentified to protect the participants’ privacy, with all identifying information removed. Access to the data was restricted to the research team, and all data were stored securely.

### Measurements

We collected and analyzed the baseline data on the sociodemographic characteristics (eg, sex, age, highest educational attainment, marital status, and family monthly income), health behaviors, mental health, physical health, and eHealth literacy of participants.

Health behaviors included physical activity levels in metabolic equivalent of task minutes (MET min) per week (measured using the International Physical Activity Questionnaire Short Form) [21], number of days of vegetable and fruit consumption in the past week, smoking status (never smoker, ex-smoker, and current smoker), intention to quit within 30 days (yes/no) for current smokers, and drinking frequency in the past year (never, monthly or less, 2-4 times per month, 2-3 times per week, and ≥4 times per week).

Mental health and perceived level of loneliness were assessed using the World Health Organization-Five (WHO-5) Well-Being Index [22] and UCLA 3-item Loneliness Scale, respectively [23]. The raw score on the WHO-5 ranges from 0 to 25, and the percentage score ranges from 0 to 100 (raw score multiplied by 4). A percentage score of 0 represents the worst possible mental health status, and 100 represents the best possible mental health status [22]. The WHO-5 has been validated for the Chinese population [24]. In our sample, the scale demonstrated good internal consistency, with a Cronbach α of 0.89. The UCLA 3-item Loneliness Scale measures 3 dimensions of loneliness (relational and social connectedness and self-perceived isolation) with a score ranging from 3 to 9, where a higher score indicates a higher level of loneliness [23]. The UCLA 3-item Loneliness Scale was validated for older Chinese adults [25]. The scale showed high reliability in the present study, with a Cronbach α of 0.88. Physical health was assessed by asking whether the participants had been medically diagnosed with each NCD (yes/no), including hypertension, diabetes, cardiovascular disease, and stroke. A multimorbidity variable was constructed (defined as having been medically diagnosed with ≥2 of the following 4 conditions: hypertension, diabetes, cardiovascular disease, and stroke).

eHealth literacy was measured using the eHealth Literacy Scale (eHEALS) [11]. The scale measures participants’ perceived skills, knowledge, and comfort toward eHealth [11]. The scale consists of 8 items, which are rated on a 5-point Likert scale ranging from 1 (strongly disagree) to 5 (strongly agree). The total score ranges from 8 to 40, with higher scores indicating higher self-perceived eHealth literacy [11]. On the basis of the score, the respondents can be categorized into 4 groups: inadequate eHealth literacy (score: 8‐15.99), low eHealth literacy (score: 16‐23.99), moderate eHealth literacy (score: 24‐31.99), and high eHealth literacy (score: 32‐40) [26]. The scale has high internal consistency, with a Cronbach α of 0.96 [26], and has been validated for Chinese older adults, with a Cronbach α of 0.88 [26].

### Statistical Analysis

Inverse probability weighting based on the sex distribution of Hong Kong older adults in 2023 (from the census) was conducted to make the sample more representative of Hong Kong’s older adult population. Descriptive statistics were used to describe the participants’ sociodemographic characteristics, eHealth literacy, health behaviors, mental health, and physical health at baseline.

The associations of sociodemographic characteristics (sex, age, highest educational attainment, family monthly income, Comprehensive Social Security Assistance [CSSA] Scheme status, type of housing, and living with family members) with the eHEALS score were analyzed using multivariable linear regression (adjusted unstandardized coefficient [b]) with mutual adjustment for baseline sociodemographic characteristics.

Multiple logistic regression (adjusted odds ratio [AOR]) was used to analyze the associations of the level of eHealth literacy with smoking and drinking status (yes/no), District Health Center member status (yes/no), multimorbidity of NCDs (yes/no), and medical diagnosis of NCDs (yes/no). Multivariable linear regression (adjusted unstandardized coefficient [b]) was used to examine the associations of the level of eHealth literacy with total physical activity (MET min/week), the WHO-5 Well-Being Index score, and the UCLA 3-item Loneliness Scale score. The associations of the level of eHealth literacy with the number of days of vegetable and fruit consumption in the past week were analyzed using Poisson regression. The models were adjusted for baseline sociodemographic characteristics. All analyses were conducted in Stata (version 15.1; StataCorp LLC). A 2-sided *P* value <.05 was considered statistically significant.

## Results

Between November 2022 and December 2024, a total of 7087 potential participants were screened and found eligible, and 6704 agreed to participate and were included in the baseline analysis. The mean time to conduct baseline assessments and deliver interventions was 102 (SD 7.3) minutes.

Table 1 shows that the mean age of the participants was 77.8 (SD 7.0) years, and 26.9% (1805/6704) of the participants were men. Most participants had primary education or below (4003/6676, 60.0%), were married (3173/6626, 47.9%), and had a family monthly income of HK $25,000 (US $3205.9) or less (5127/6653, 77.1%). Moreover, 25.4% (1691/6650) of participants were supported by the CSSA Scheme. Most participants were retired (6460/6694, 96.5%), living in public housing (4829/6696, 72.1%), and living with family members (3479/6696, 52.0%). In total, 71.2% (4774/6704) of participants were not District Health Center members. The mean eHEALS score of participants was 18.2 (SD 10.2), and most participants (2897/6566, 44.1%) had inadequate eHealth literacy (score: 8‐15.99).

**Table 1.** Sociodemographic characteristics of participants (N=6704)^a^.

Characteristic	Participants, n (%)
Sex	
Male	1805 (26.9)
Female	4894 (73.1)
Age^b^ (years)	77.8 (7.0)
Highest educational attainment	
Primary education or below	4003 (60)
Secondary education	2288 (34.2)
Tertiary education	385 (5.8)
Marital status	
Single	397 (6)
Married	3173 (47.9)
Divorced	454 (6.8)
Widowed	2602 (39.3)
Family monthly income	
None	204 (3.1)
HK $25,000 (US $ 3205.9) or less	5127 (77.1)
More than HK $25,000 (US $ 3205.9)	1322 (19.9)
Support from the Comprehensive Social Security Assistance Scheme	
No	4959 (74.6)
Yes	1691 (25.4)
Occupational status	
Full-time (employed)	35 (0.5)
Part-time (employed)	90 (1.3)
Unemployed	13 (0.2)
Retired	6460 (96.5)
Caregiver/housewife	96 (1.4)
Type of housing	
Public	4829 (72.1)
Private	1546 (23.1)
Other	321 (4.8)
Living with family members	
No	3046 (45.5)
Yes	3479 (52)
Other	171 (2.5)
District Health Center member status	
No	4774 (71.2)
Yes	1930 (28.8)
eHealth literacy (eHEALS score^b^: 8‐40)^c^	18.2 (10.2)
Inadequate (score: 8‐15.99)	2897 (44.1)
Low (score:16‐23.99)	1322 (20.1)
Moderate (score: 24‐31.99)	1182 (18)
High (score: 32‐40)	1165 (17.7)

a The proportions were weighted by sex distribution of older adults in Hong Kong 2023. The observations (n) were unweighted.

b Reported as mean (SD).

c eHealth literacy is measured on a 5-point eHealth Literacy Scale (eHEALS), with ratings ranging from 1 (strongly disagree) to 5 (strongly agree). The overall score ranges from 8 to 40, with a higher score indicating more perceived skills in finding, evaluating, and using electronic information to make health decisions.

Table 2 shows that the participants’ mean total physical activity was 628.7 (SD 542.9) MET min per week. The mean number of days on which vegetables and fruits were consumed in the past week was 6.6 (SD 1.3) and 5.9 (SD 2.0), respectively. In total, 3% (198/6686) of participants were current smokers, and 91% (163/179) had no intention to quit. Moreover, 8.5% (570/6704) of participants were current drinkers. The mean percentage score on the WHO-5 Well-being Index was 68.1 (SD 21.3), and the mean score on the UCLA 3-item Loneliness Scale was 4.0 (SD 1.6). Notably, 45.2% (3020/6674) of participants had multimorbidity of NCDs (medically diagnosed with ≥2 NCDs). Of the 6704 participants, 60.5% (n=4059) were medically diagnosed with hypertension, 27.1% (n=1816) had diabetes, 13.3% (n=894) had cardiovascular disease, and 4.6% (n=309) had a medical diagnosis of stroke.

**Table 2.** Health behaviors, mental health, and physical health of participants (N=6704)^a^.

Parameter	Outcome
Physical activity, IPAQ-SF continuous score (MET^b^ min/week), mean (SD)^c^	
Walking	463.5 (443.8)
Moderate	139.9 (222.6)
Vigorous	25.2 (108.6)
Total	628.7 (542.9)
Number of days of vegetable consumption in the past week, mean (SD)	6.6 (1.3)
Number of days of fruit consumption in the past week, mean (SD)	5.9 (2.0)
Smoking status, n (%)	
Never smoker	5812 (86.9)
Ex-smoker	676 (10.1)
Current smoker	198 (3)
Intention to quit for current smokers, n (%)	
No	163 (91)
Yes	16 (9)
Drinking frequency in the past year, n (%)	
Never	6104 (91.5)
Monthly or less	289 (4.3)
2‐4 times per month	129 (1.9)
2‐3 times per week	46 (0.7)
>4 times per week	106 (1.6)
WHO-5^d^ Well-Being Index percentage score (0‐100)^e^, mean (SD)	68.1 (21.3)
UCLA 3-item Loneliness Scale score: 3‐9)^f^, mean (SD)	4.0 (1.6)
Multimorbidity^g^ of NCDs^h^, n (%)	
No	3654 (54.7)
Yes	3020 (45.2)
Medical diagnosis of hypertension, n (%)	
No	2645 (39.5)
Yes	4059 (60.5)
Medical diagnosis of diabetes, n (%)	
No	4888 (72.9)
Yes	1816 (27.1)
Medical diagnosis of cardiovascular disease, n (%)	
No	5810 (86.7)
Yes	894 (13.3)
Medical diagnosis of stroke, n (%)	
No	6395 (95.4)
Yes	309 (4.6)

a The proportions were weighted by sex distribution of older adults in Hong Kong 2023. The observations (n) were unweighted.

b MET: metabolic equivalent of task.

c The International Physical Activity Questionnaire Short Form (IPAQ-SF) continuous scores are expressed in MET min/week: MET level × minutes of activity × events per week. The total physical activity is computed as the sum of walking, moderate, and vigorous scores (MET min/week).

d WHO-5: World Health Organization-Five Well-Being Index.

e The raw score, ranging from 0 to 25, is multiplied by 4 to obtain a percentage score. A percentage score of 0 represents the worst possible quality of life, whereas a score of 100 represents the best possible quality of life.

f The 3-point response scale for each item ranges from “hardly ever or never” (1 point) to “often” (3 points), and the total score is the sum of all items, which ranges from 3 to 9, with higher scores indicating a higher level of perceived loneliness.

g Multimorbidity was defined as having been medically diagnosed with ≥2 of the following 4 conditions: hypertension, diabetes, cardiovascular disease, and stroke.

h NCD: noncommunicable disease.

Table 3 shows that increasing age (adjusted b −0.32, 95% CI −0.35 to −0.28; *P*<.001), support from the CSSA Scheme (adjusted b −1.49, 95% CI −2.04 to −0.95; *P*<.001), and living in public housing (adjusted b −1.60, 95% CI −2.69 to −0.50; *P*=.004) were associated with a lower eHEALS score, after mutual adjustment for baseline sociodemographic characteristics. Secondary education (adjusted b 4.31, 95% CI 3.81-4.82; *P*<.001) and tertiary education (adjusted b 9.04, 95% CI 8.04-10.06; *P*<.001) were associated with higher eHEALS scores.

**Table 3.** Associations of sociodemographic characteristics with eHealth literacy of participants (N=6704).

Characteristic	Association with eHealth Literacy Scale score (8‐40)			
	Crude b (95% CI)	*P* value	Adjusted b (95% CI)^a^	*P* value
Sex (reference: male)				
Female	0.05 (−0.50 to 0.61)	.85	0.17 (−0.38 to 0.71)	.89
Age	−0.40 (−0.43 to −0.36)	<.001	−0.32 (−0.35 to −0.28)	<.001
Highest educational attainment (reference: primary education or below)				
Secondary education	5.37 (4.86 to 5.87)	<.001	4.31 (3.81 to 4.82)	<.001
Tertiary education	9.05 (8.02 to 10.08)	<.001	9.04 (8.04 to 10.06)	<.001
Family monthly income (reference: none)				
HK $25,000 (US $3205.9) or less	0.50 (−0.12 to 1.12)	.12	0.24 (−0.34 to 0.82)	.16
More than HK $25,000 (US $3205.9)	2.27 (0.81 to 3.73)	.002	0.91 (−0.46 to 2.29)	.06
Support from the Comprehensive Social Security Assistance Scheme (reference: no)				
Yes	−2.39 (−2.96 to −1.82)	<.001	−1.49 (−2.04 to −0.95)	<.001
Type of housing (reference: other)				
Public	−2.77 (−3.93 to −1.62)	<.001	−1.60 (−2.69 to −0.50)	.004
Private	0.24 (−0.98 to 1.47)	.70	0.13 (−0.73 to 1.41)	.69
Living with family members (reference: other)				
No	0.06 (−1.72 to 1.84)	.94	−0.79 (−2.46 to 0.87)	.87
Yes	0.99 (−0.78 to 2.76)	.95	−0.43 (−2.10 to 1.23)	.89

a The model was mutually adjusted for all sociodemographic characteristics listed in the table, including sex, age, highest educational attainment, family monthly income, Comprehensive Social Security Assistance status, type of housing, and living with family members.

Descriptive analysis showed that 93% (6227/6704) of participants were smartphone users. In total, 57.5%(3549/6173) used a smartphone for 1 hour or more daily, 91.5% (6135/6704) had instant messaging apps installed on the smartphone, and 60.2% (3992/6634) had not searched for health-related information from online sources using the smartphone (Multimedia Appendix 1). Table 4 shows that participants with moderate eHealth literacy (score: 24‐31.99) were less likely to be current smokers (AOR 0.60, 95% CI 0.38-0.95; *P*=.04) and more likely to be District Health Center members (AOR 1.52, 95% CI 1.30-1.77; *P*<.001), and moderate eHealth literacy was associated with higher total physical activity levels (adjusted b 39.83, 95% CI 2.04-77.62; *P*=.04) and a higher number of days of fruit consumption in the past week (adjusted b 0.034, 95% CI 0.006-0.063; *P*=.04). Participants with high eHealth literacy (score 32‐40) were less likely to be current smokers (AOR 0.57, 95% CI 0.36-0.92; *P*=.03) and more likely to be District Health Center members (AOR 1.76, 95% CI 1.51-2.05; *P*<.001), and high eHealth literacy was associated with a higher number of days of fruit consumption in the past week (adjusted b 0.046, 95% CI 0.017-0.075; *P*=.003).

**Table 4.** Associations of eHealth literacy with health behaviors of participants (N=6704).

Health behavior	eHealth literacy (eHEALS^a^ score: 8‐40) (reference: inadequate eHealth literacy [score: 8-15.99], n=2897)					
	Low (score:16‐23.99) n=1322	*P* value	Moderate (score: 24‐31.99) n=1182	*P* value	High (score: 32‐40) n=1165	*P* value
Current smoker						
Crude OR (95% CI)	0.94 (0.65 to 1.35)	.73	0.69 (0.45 to 1.06)	.09	0.66 (0.42 to 1.02)	.06
Adjusted OR (95% CI)^b^	0.90 (0.61 to 1.34)	.67	0.60 (0.38 to 0.95)	.04	0.57 (0.36 to 0.92)	.03
Current drinker						
Crude OR (95% CI)	1.23 (0.98 to 1.55)	.07	1.33 (1.06 to 1.68)	.02	1.01 (0.79 to 1.30)	.91
AOR (95% CI)^b^	1.16 (0.92 to 1.48)	.10	1.19 (0.93 to 1.53)	.06	0.85 (0.65 to 1.11)	.93
Physical activity (total MET min/week)						
Crude b (95% CI)	5.85 (−29.77 to 41.47)	.75	74.47 (37.54 to 111.39)	<.001	80.12 (42.96 to 117.28)	<.001
Adjusted b (95% CI)^b^	−10.07 (−45.96 to 25.82)	.80	39.83 (2.04 to 77.62)	.04	36.51 (−2.16 to 75.18)	.06
District Health Center member status						
Crude OR (95% CI)	1.25 (1.07 to 1.45)	.003	1.87 (1.61 to 2.16)	<.001	2.27 (1.97 to 2.63)	<.001
AOR (95% CI)^c^	1.13 (0.97 to 1.32)	.06	1.52 (1.30 to 1.77)	<.001	1.76 (1.51 to 2.05)	<.001
Number of days of vegetable consumption in the past week^d^						
Crude b (95% CI)	0.01 (−0.02 to 0.03)	.68	0.01 (−0.01 to 0.04)	.72	0.02 (−0.01 to 0.05)	.44
Adjusted b (95% CI)^b^	0.01 (−0.02 to 0.03)	.70	0.02 (−0.01 to 0.04)	.83	0.02 (−0.01 to 0.05)	.45
Number of days of fruit consumption in the past week^d^						
Crude b (95% CI)	−0.003 (−0.030 to 0.024)	.56	0.034 (0.006 to 0.062)	.04	0.047 (0.019 to 0.074)	.003
Adjusted b (95% CI)^b^	−0.006 (−0.033 to 0.022)	.57	0.034 (0.006 to 0.063)	.04	0.046 (0.017 to 0.075)	.003

a eHEALS: eHealth Literacy Scale.

b Adjusted for sex, age, highest educational attainment, family monthly income, living with family members, and being a District Health Center member.

c Adjusted for sex, age, highest educational attainment, family monthly income, and living with family members.

d Poisson regression was used to calculate the unstandardized coefficient.

Table 5 shows that participants with moderate eHealth literacy (score 24‐31.99) were less likely to have a medical diagnosis of diabetes (AOR 0.73, 95% CI 0.62-0.85; *P*<.001) and stroke (AOR 0.58, 95% CI 0.40-0.85; *P*=.004), and moderate eHealth literacy was associated with a higher score on the WHO-5 Well-being Index (adjusted b 2.89, 95% CI 1.42-4.36; *P*<.001) and a lower score on the UCLA 3-item Loneliness Scale (adjusted b −0.26, 95% CI −0.37 to −0.15; *P*<.001). Participants with high eHealth literacy (score: 32‐40) were less likely to have a medical diagnosis of hypertension (AOR 0.77, 95% CI 0.67-0.90; *P*=.004) and diabetes (AOR 0.69, 95% CI 0.58-0.81; *P*<.001), and high eHealth literacy was associated with a higher score on the WHO-5 Well-being Index (adjusted b 5.37, 95% CI 3.86-6.87; *P*<.001) and lower score on the UCLA 3-item Loneliness Scale (adjusted b −0.34, 95% CI −0.45 to −0.23; *P*<.001).

**Table 5.** Associations of eHealth literacy with the physical health and mental health of participants (N=6704).

Parameter	eHealth literacy (eHEALS^a^ score: 8‐40) (reference: inadequate eHealth literacy [score: 8-15.99], n=2897)					
	Low (score:16‐23.99) n=1322	*P* value	Moderate (score: 24‐31.99) n=1182	*P* value	High (score: 32‐40) n=1165	*P* value
Medical diagnosis of hypertension						
Crude OR^b^ (95% CI)	1.01 (0.88 to 1.15)	.92	0.77 (0.67 to 0.89)	<.001	0.65 (0.56 to 0.74)	<.001
Adjusted OR (95% CI)^c^	1.06 (0.93 to 1.22)	.95	0.89 (0.77 to 1.02)	.06	0.77 (0.67 to 0.90)	.004
Medical diagnosis of diabetes						
Crude OR (95% CI)	0.84 (0.73 to 0.97)	.02	0.73 (0.63 to 0.85)	<.001	0.69 (0.59 to 0.80)	<.001
Adjusted OR (95% CI)^c^	0.83 (0.71 to 0.96)	.03	0.73 (0.62 to 0.85)	<.001	0.69 (0.58, 0.81)	<.001
Medical diagnosis of cardiovascular disease						
Crude OR (95% CI)	0.99 (0.83 to 1.20)	.98	0.81 (0.66 to 1.00)	.06	0.93 (0.76 to 1.14)	.49
Adjusted OR (95% CI)^c^	1.10 (0.91 to 1.34)	.98	0.97 (0.79 to 1.21)	.07	1.12 (0.91 to 1.40)	.52
Medical diagnosis of stroke						
Crude OR (95% CI)	0.76 (0.55 to 1.04)	.08	0.58 (0.40 to 0.83)	.003	0.77 (0.55 to 1.06)	.11
Adjusted OR (95% CI)^c^	0.74 (0.54 to 1.03)	.08	0.58 (0.40 to 0.85)	.004	0.80 (0.56 to 1.12)	.13
Multimorbidity^f^ of NCDs^g^						
Crude b (95% CI)	0.83 (0.57 to 1.09)	.12	0.67 (0.44 to 1.07)	.10	0.65 (0.50 to 1.12)	.14
Adjusted b (95% CI)^c^	0.86 (0.62 to 1.12)	.14	0.69 (0.48 to 1.10)	.10	0.66 (0.52 to 1.14)	.14
WHO-5 Well-Being Index score^d^						
Crude b (95% CI)	0.96 (−0.42 to 2.34)	.17	2.75 (1.32 to 4.19)	<.001	5.65 (4.21 to 7.09)	<.001
Adjusted b (95% CI)^c^	0.87 (−0.52 to 2.27)	.15	2.89 (1.42 to 4.36)	<.001	5.37 (3.86 to 6.87)	<.001
UCLA 3-item Loneliness Scale score^e^						
Crude b (95% CI)	−0.19 (−0.29 to −0.08)	.003	−0.27 (−0.38 to −0.15)	<.001	−0.40 (−0.52 to −0.29)	<.001
Adjusted b (95% CI)^c^	−0.17 (−0.28 to −0.07)	.003	−0.26 (−0.37 to −0.15)	<.001	−0.34 (−0.45 to −0.23)	<.001

a eHEALS: eHealth Literacy Scale.

b OR: odds ratio.

c Adjusted for sex, age, highest educational attainment, family monthly income, living with family members, and District Health Center member.

d WHO-5: World Health Organization-Five Well-Being Index. The score ranges from 0 to 25, with 0 representing the worst possible and 25 representing the best possible quality of life. The score is multiplied by 4. A percentage score of 0 represents the worst possible quality of life, whereas a score of 100 represents the best possible quality of life.

e The 3-point response scale for each item ranges from “hardly ever or never” (1 point) to “often” (3 points), and the total score is the sum of all items, which ranges from 3 to 9, with higher scores indicating a higher level of perceived loneliness.

f Multimorbidity was defined as having been medically diagnosed with ≥2 of the following 4 conditions: hypertension, diabetes, cardiovascular disease, and stroke.

g NCD: noncommunicable disease.

## Discussion

### Principal Findings

We analyzed the baseline data from an ongoing, large-scale, quasi-experimental, pre-post study to investigate the level of eHealth literacy and its associations with health behaviors, primary care service use, mental health, and physical health among Hong Kong Chinese older adults (N=6704). The results suggested that their eHealth literacy level was generally low. In addition, our findings showed that older adults with higher eHealth literacy were associated with positive health behaviors and better health-related outcomes at baseline. A key strength of this study is its examination of specific, modifiable health behaviors (eg, smoking, physical activity, and diet) and health outcomes (eg, mental health and NCD risks). This granular approach provides more actionable insights for public health interventions. For example, identifying a link between eHealth literacy and smoking status allows for more targeted health promotion campaigns. This specificity moves the field beyond broad associations toward evidence that can directly inform the content of programs that leverage digital tools for health improvement in aging populations.

The mean eHealth literacy score of the study participants was 18.2 (SD 10.2), which was lower than the scores reported in the United States (30.9) [16], South Korea (30.5) [17], Norway (25.7) [18], and China (21.4) [19]. The notably higher age (mean 77.8, SD 7.0 years) of our sample is likely a key contributor to this low average score, a conclusion supported by our finding that increasing age was the most significant demographic predictor of lower eHealth literacy. This suggests that interventions to enhance older adults’ eHealth literacy are much needed. Our study indicated that 44.1% of older adults (mean age 77.8, SD 7.0 years) lacked eHealth literacy despite having a smartphone with internet access. This can be attributed to older adults’ attitudes toward the internet, including self-efficacy in managing digital technology and a preference for in-person interaction with health practitioners [27]. eHealth literacy may also be lower due to age-related problems and cognitive decline [27]. These findings are relevant to delivering eHealth resources to older adults [27]. Overcoming these sociodemographic and psychological barriers may increase eHealth literacy and help older adults improve their general health. In addition, despite 93% (6227/6697) of participants owning a smartphone, 60.2% (3992/6634) reported never having used it to search for health information (Multimedia Appendix 1). This highlights a critical gap between device ownership and meaningful health-related use, underscoring that access to technology alone is insufficient. This “know-do” gap underscores the urgent need for interventions that build skills and confidence in searching for health information with technological devices among older adults.

**Figure 1.**
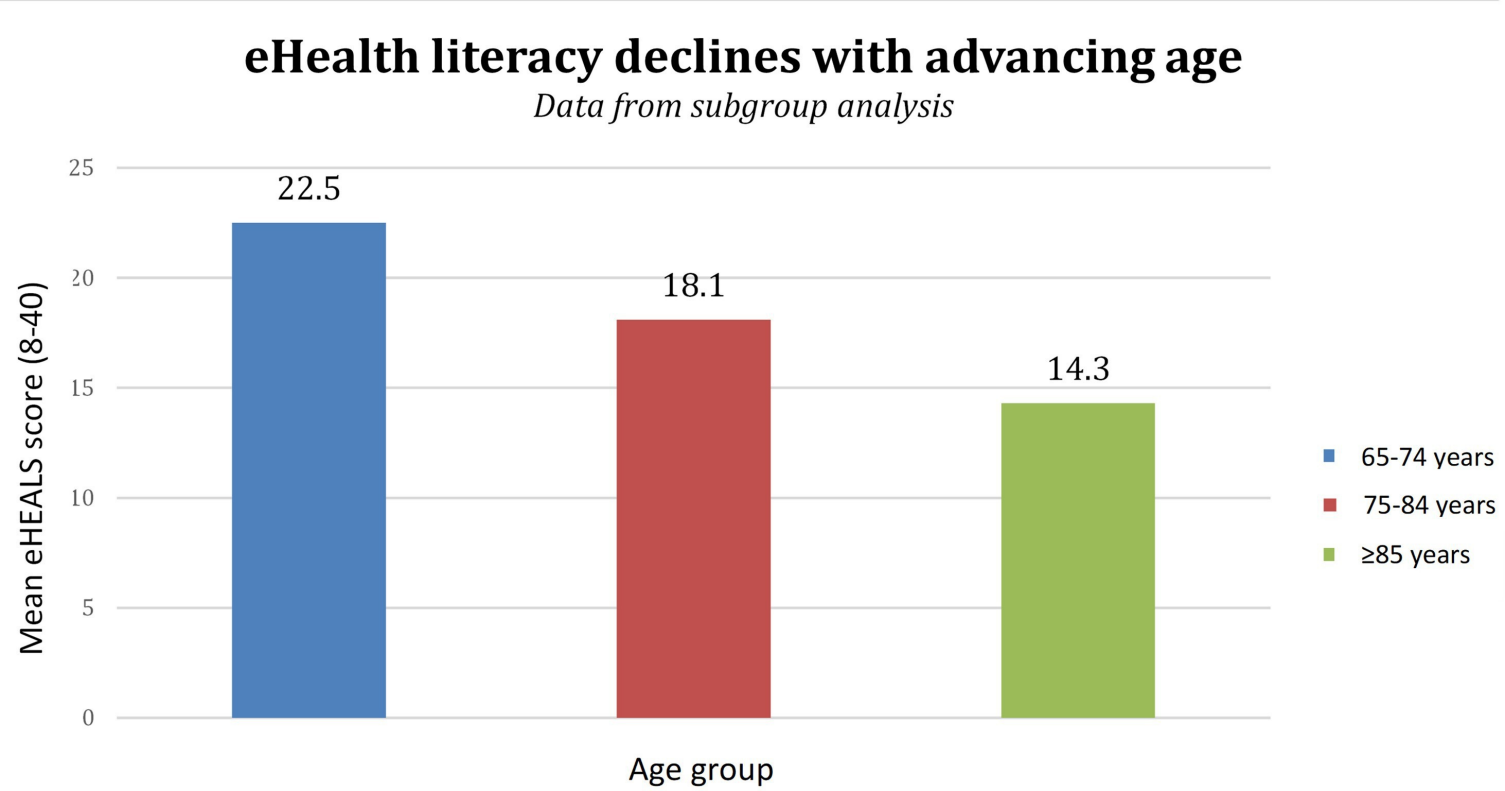
Mean eHealth literacy Scale (eHEALS) score by age group in the study sample (N=6704).

Furthermore, we performed subgroup analyses of eHealth literacy by age group (65‐74 years, 75‐84 years, and ≥85 years) in our study sample (Figure 1). The results showed a stepwise decline in eHealth literacy across age strata. Older adults with increasing age had the highest burden of chronic disease and the greatest need for health care services, yet they are the most likely to be excluded from an increasingly digitized health care system. Our study’s findings can serve as a data point for policymakers and health care providers, highlighting the urgent need for tailored, age-appropriate support systems to prevent the digital divide from becoming a health equity crisis.

Our findings showed that older adults with higher education were associated with higher eHealth literacy, and older adults with indicators of lower socioeconomic status (receiving support from the CSSA Scheme and living in public housing) were associated with lower eHealth literacy. Lower-income older adults may have poorer access to digital technology, such as limited computer and internet access, leading to skill disparity [28]. The relationship between socioeconomic status and eHealth literacy needs to be investigated in depth to prevent the potential loss of the silver economy in a digitalized society. It is important to consider the social disparities and the digital divide factors when promoting eHealth services to ensure that those needing these services are not left out [28]. Primary health care is the cornerstone of the health care system, and we should optimize the primary health care services to enhance older adults’ eHealth literacy. In addition, training should be provided to carers and the younger generation (eg, university students) to guide underprivileged older adults in using electronic information technology and meeting their health needs in the digital age.

We found that older adults with moderate and high eHealth literacy were less likely to be smokers and more likely to engage in positive health behaviors. Higher eHealth literacy may enhance an individual’s self-efficacy in managing their health. With an enhanced ability to find, evaluate, and apply online health information, older adults may feel more confident in making informed decisions [29], leading to better health behaviors such as improved diet or increased physical activity and more effective self-management of chronic conditions. In addition, greater access to eHealth resources increases the awareness of health maintenance and encourages older adults to adopt healthy lifestyles [30]. Older adults skilled in using digital health services will be able to communicate with health care professionals in advance and manage their health more efficiently, enabling them to receive more comprehensive NCD prevention services.

Our findings revealed a strong association between higher eHealth literacy and better mental health outcomes, specifically higher levels of well-being and lower levels of loneliness. This finding aligns with a growing body of literature demonstrating the positive role of digital engagement in the psychosocial health of older adults [31]. For instance, a study in Hong Kong during the COVID-19 pandemic found that the use of instant messaging apps was a key factor in mitigating loneliness [32]. Our results expand on this by suggesting that eHealth literacy, as a core component of digital competency, is a crucial enabler of such beneficial social connections. The mechanism is likely 2-fold. First, digitally literate older adults can more easily use technology to maintain contact with family and friends, thereby strengthening their social support networks. Second, those with low eHealth literacy may struggle to differentiate credible information from misinformation online [33], leading to increased anxiety and negative emotional states. The implication for practice is significant: interventions aimed at improving mental health in older adults should consider incorporating digital literacy training not merely as a technical skill but as a fundamental tool for fostering social connectedness and resilience in a digital world.

### Implications and Future Directions

Based on the findings identified in this cross-sectional study, several avenues for future research are warranted to deepen our understanding and inform effective interventions. First, there is a critical need for longitudinal studies to establish temporal relationships between eHealth literacy and health outcomes. For instance, a recent 3-wave longitudinal study demonstrated that higher baseline eHealth literacy predicted a better health-promoting lifestyle over time among Chinese older adults [34]. Future research should build on this finding by following up older adults to determine whether improving eHealth literacy leads to sustained health behavior change and better long-term health outcomes and to explore the mediating pathways (eg, self-efficacy) in these relationships.

Second, to move beyond association and test for causality, future work should include intervention studies, such as randomized controlled trials. These studies could evaluate the effectiveness of tailored eHealth literacy training programs on specific outcomes, including health behavior change, chronic disease self-management, and mental well-being in older adults. Such interventions should be multifaceted and co-designed with older adults, moving beyond basic technology access to build practical skills and confidence. Strategies could include (1) tailored, hands-on training workshops; (2) intergenerational peer-tutoring models; (3) the development of age-friendly user interfaces for health apps; and (4) the integration of eHealth literacy support within primary care settings and community centers.

Third, the measurement of eHealth literacy itself requires advancement. This study, similar to many others, relied on a self-report scale. Future research would benefit from incorporating objective, performance-based assessments. This would provide a more accurate measure of actual competency and help bridge the gap between perceived and demonstrated skills. Additionally, research is needed to validate eHealth literacy assessment tools and their cut-off scores for the older and oldest older populations.

Finally, to enhance generalizability, future research should include older adults with a history of mental illness. While they were excluded from our analysis to avoid confounding the mental well-being outcomes, research focus on this vulnerable group is essential for developing tailored interventions that address their specific barriers to digital engagement and health management [35], thus further narrowing the digital divide.

### Limitations

This study has several limitations. First, the findings were based on an analysis of the baseline data, and causal relationships between variables could not be confirmed. Given the initial observational data, intervention studies (eg, randomized controlled trials) should be conducted to determine causal relationships between eHealth literacy, health behaviors, and health-related outcomes. Follow-up data and qualitative research may provide more insights into these mechanisms in the future. Second, our study excluded older adults with a self-reported history of mental illness (eg, depression and anxiety), and this exclusion may limit the generalizability of our findings. Mental health status may be associated with an individual’s motivation and ability to engage with digital technology [31]. Further research is needed to investigate the relationships between eHealth literacy and health outcomes, specifically within this vulnerable subgroup of older adults. Third, individual eHealth literacy and health status are closely related to the local economic development level [36], and the study findings may not be generalizable to regions with different socioeconomic contexts, cultural backgrounds, or levels of digital infrastructure. In addition, the study was conducted in Hong Kong, a high-income region with advanced digital infrastructure. The observed levels of eHealth literacy and their associations with health outcomes may not be directly generalizable to older adults in low-income or middle-income settings, where access to technology and digital skills may be substantially different. Fourth, this study used the eHEALS to assess eHealth literacy. While widely used and validated, this scale has significant limitations. Developed in 2006 [11], eHEALS may not fully capture the complex skills required to navigate today’s ecosystem of mobile apps and interactive health technologies [37]. Furthermore, newer instruments such as the Digital Health Literacy Instrument (DHLI) have been developed and show good utility in Chinese older adults [38]. Recent systematic reviews also highlight the need for more robust, performance-based tools, as eHEALS assesses self-perceived skills rather than actual competence, which may be subject to self-report bias [37,39]. Finally, the generalizability of our findings to the entire older adult population in Hong Kong may be limited. While our sample was large and weighted by sex distribution to match the Hong Kong census, the mean age of our participants was 77.8 (SD 7.0) years, which is higher than that of the general older adult population in Hong Kong [40].

### Conclusions

eHealth literacy is an essential skill in a rapidly digitalizing world. The findings showed that the eHealth literacy of older adults in Hong Kong was low and needs improvement, especially in the context of global primary health care reform. We also observed that higher levels of eHealth literacy were associated with health-promoting behaviors, primary care service use, and better physical and mental health outcomes. With the application of the results from this study, tailored interventions should be implemented to improve eHealth literacy and narrow the digital divide among older adults.

## Supplementary material

10.2196/74110 Multimedia Appendix 1 Characteristics of smartphone usage of participants (N=6704).

10.2196/74110 Checklist 1 STROBE (Strengthening the Reporting of Observational Studies in Epidemiology) checklist.

## Abbreviations

AOR: adjusted odds ratio
CSSA: Comprehensive Social Security Assistance
DHLI: Digital Health Literacy Instrument
eHEALS: eHealth Literacy Scale
MET: metabolic equivalent of task
NCD: noncommunicable disease
OR: odds ratio
WHO-5: World Health Organization-Five Well-Being Index
