# Supplementary material for: Level of eHealth Literacy and Its Associations With Health Behaviors and Outcomes in Chinese Older Adults: Cross-Sectional Analysis of Baseline Data From a Large-Scale Community Project
Source: JMIR Aging. 2026 Jan 30;9:e74110. doi: 10.2196/74110 (PMC12857900; doi:10.2196/74110)
Supplement: Multimedia Appendix 1 [file aging-v9-e74110-s001.docx]

**Appendix**

**Appendix Table 1. Characteristics of smartphone usage of participants (n = 6704) ^a.^**

1. The proportions were weighted by sex distribution of older adults in Hong Kong 2023. The observations (n) were unweighted.

|  | **n (%)** |
| --- | --- |
| **Smartphone user** |  |
| No | 470 (7.0) |
| Yes | 6227 (93.0) |
| **Daily time spent using the smartphone** |  |
| None | 65 (1.0) |
| Less than 1 hour | 2559 (41.4) |
| 1-2 hours | 1746 (28.3) |
| 2-3 hours | 777 (12.6) |
| 3-4 hours | 415 (6.7) |
| Above 4 hours | 611 (9.9) |
| **Do you have instant messaging apps installed on your smartphone?** |  |
| No | 569 (8.5) |
| Yes | 6135 (91.5) |
| **Have you searched for health-related information from online sources?** |  |
| No | 3992 (60.2) |
| Yes | 2642 (39.8) |
